# Remimazolam in older surgical patients: postoperative delirium, hemodynamic stability, and mechanistic considerations

**DOI:** 10.3389/fmed.2026.1942031

**Published:** 2026-10-22

**Authors:** Donglan Peng, Zhaoying Zhong, Ping Zou, Lei Yuan, Caihong Song, Bin Zhou, Baozhen Liao, Shumei Wang, Jianqiang Zeng

**Affiliations:** Department of Anesthesiology, Ganzhou Cancer Hospital, Ganzhou, Jiangxi, China

**Keywords:** emergence delirium, geriatric anesthesia, hemodynamic stability, older adults, postoperative delirium, remimazolam, surgery

## Abstract

The growing number of older adults undergoing surgery has increased interest in anesthetic strategies that preserve physiological stability without worsening postoperative neurocognitive outcomes. Remimazolam is an ultra-short-acting benzodiazepine that produces hypnosis through positive allosteric modulation of the *γ*-aminobutyric acid type A (GABA-A) receptor and is rapidly hydrolyzed to the low-activity metabolite CNS7054. This focused narrative review examines remimazolam in older surgical patients, with particular emphasis on postoperative delirium (POD) and hemodynamic stability. Randomized trials and meta-analyses generally report fewer hypotensive events with remimazolam than with propofol, but neurocognitive findings are heterogeneous. Earlier trials in orthopedic, hip, gastrectomy, and abdominal surgery largely reported similar POD incidence, and one trial in frail patients undergoing hip surgery reported a lower POD incidence with remimazolam. In contrast, a 2026 randomized non-inferiority trial involving 455 patients aged ≥65 years undergoing lung resection found a higher overall incidence of delirium with remimazolam (48% vs. 31%), driven predominantly by emergence delirium in the post-anesthesia care unit (46% vs. 28%), whereas delirium after post-anesthesia care unit discharge through postoperative day 3 was similar (4% vs. 5%). This temporal distinction indicates that outcome definitions and assessment windows materially influence apparent treatment effects. PACU delirium is also associated with subsequent adverse outcomes in observational studies, underscoring the clinical importance of this early phenotype. The relatively consistent hemodynamic advantage of remimazolam should therefore be regarded separately from neurocognitive benefit; reduced hypotension has not yet been shown to translate into delirium prevention. Preclinical anti-inflammatory and organ-protective pathways are biologically plausible but remain unconfirmed at clinically relevant neurocognitive endpoints. Current evidence supports individualized use rather than routine selection of remimazolam for delirium prevention.

## 1 Introduction

Population aging is reshaping perioperative medicine. Older surgical patients frequently have frailty, multimorbidity, reduced physiological reserve, and baseline cognitive vulnerability, which increase susceptibility to hypotension and postoperative delirium (POD) (1, 2). POD is associated with prolonged hospitalization, functional decline, and mortality. Anesthetic choice may therefore represent one modifiable component of a broader geriatric perioperative strategy, but any drug effect must be interpreted alongside pain, hypoxemia, infection, sleep disruption, and baseline vulnerability.

Recent European guidance emphasizes risk assessment, validated repeated screening, and multicomponent prevention of postoperative delirium, including assessment beginning in the recovery room or by postoperative day 1 (3). Recommendations for perioperative brain health likewise favor a coordinated approach to preoperative cognitive vulnerability, informed consent, and postoperative follow-up rather than assuming that a single anesthetic choice ensures neurocognitive safety (4).

Propofol remains a widely used intravenous hypnotic because of its rapid onset and favorable recovery profile, but dose-dependent vasodilation and cardiovascular depression can be consequential in older adults (5). Remimazolam retains benzodiazepine sedative-amnestic effects but undergoes rapid ester hydrolysis to the low-activity metabolite CNS7054; this rapid metabolism facilitates predictable offset and clinical titration (6–8). Longer-acting benzodiazepines remain a concern in vulnerable older adults because of prolonged sedation and delirium risk (9). These differences motivate the question of whether remimazolam can improve cardiovascular tolerability without worsening neurocognitive outcomes.

The central clinical question is therefore not simply whether remimazolam is an effective hypnotic, but how its benefits and risks should be interpreted in older surgical patients. The most reproducible comparative signal is less hypotension than with propofol, whereas POD findings are heterogeneous and have become more controversial with recent randomized evidence (10–13). Accordingly, this review focuses on two linked but distinct outcomes—POD, including emergence delirium, and hemodynamic stability—while using respiratory and procedural-sedation data only as contextual safety evidence.

### 1.1 Scope and literature update

This focused narrative review was updated through 2 October 2026 using PubMed/MEDLINE and citation tracking of key trials and reviews. Searches combined “remimazolam” with terms including “older” or “elderly,” “surgery,” “postoperative delirium,” “emergence delirium,” “propofol,” “hypotension,” and “randomized controlled trial.” Priority was given to randomized trials in older surgical populations, recent systematic reviews/meta-analyses, and mechanistic studies directly relevant to remimazolam. Because the article was conceived as a narrative review and no prospective systematic-review protocol was registered, we did not reclassify it as a systematic review or perform a *de novo* meta-analysis.

## 2 Pharmacological basis of remimazolam

### 2.1 GABA-A receptor modulation and sedative-amnestic effects

Remimazolam is an ultra-short-acting benzodiazepine that positively modulates GABA-A receptors, enhancing endogenous GABA-mediated inhibition and producing hypnosis, anxiolysis, and anterograde amnesia (6, 7) (Figure 1). GABAergic sedation can still contribute to oversedation and respiratory depression, particularly with opioids or other central nervous system depressants; short duration and titratability therefore do not eliminate the need for careful monitoring (14).

**Figure 1:**
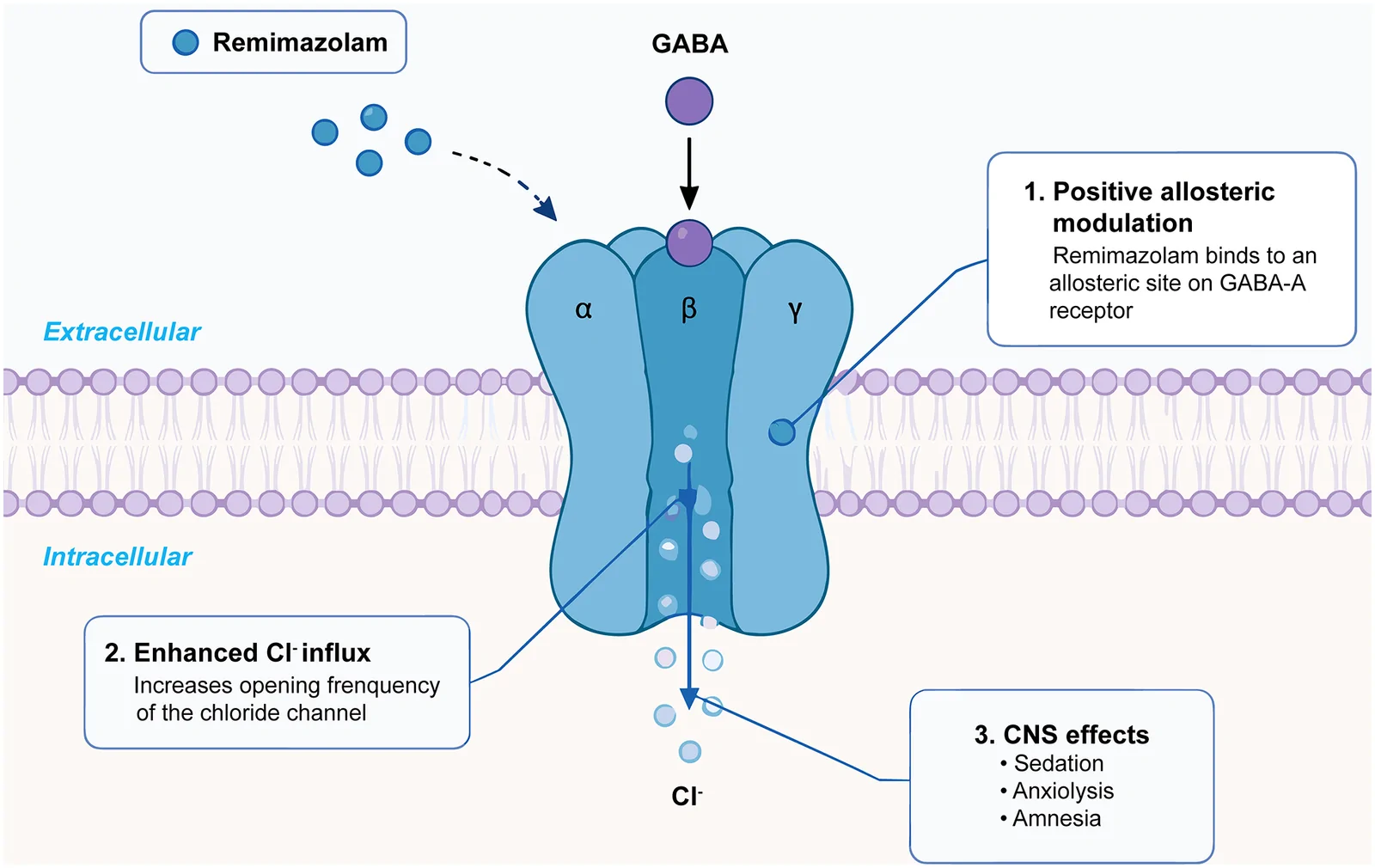
Pharmacological mechanisms and pharmacokinetic characteristics of remimazolam in older surgical patients. Remimazolam is an ultra-short-acting benzodiazepine that produces hypnosis through positive allosteric modulation of the *γ*-aminobutyric acid type A (GABA-A) receptor. It is rapidly hydrolyzed by carboxylesterase 1 (CES1) to the low-activity metabolite CNS7054, limiting accumulation and facilitating recovery. Its effects can be antagonized by flumazenil. These pharmacological properties support titratability and rapid offset but do not, by themselves, establish protection from postoperative delirium or other end-organ complications.

### 2.2 Soft-drug design and metabolism

A defining feature of remimazolam is its metabolically labile ester linkage. Rapid carboxylesterase-mediated hydrolysis produces CNS7054, a metabolite with much lower activity, and supports high clearance, a short context-sensitive half-time, and limited dependence on cytochrome P450 metabolism (6, 7, 15). Rapid metabolism facilitates predictable offset and limits prolonged accumulation after discontinuation.

Age alone may not markedly change remimazolam disposition, but frailty, hepatic dysfunction, body composition, altered pharmacodynamic sensitivity, and concomitant opioid exposure can modify clinical response. Conservative titration remains appropriate in older adults (7, 15).

### 2.3 Reversibility with flumazenil

Like midazolam and other benzodiazepines, remimazolam can be antagonized with flumazenil; reversibility therefore distinguishes it from propofol but not from conventional benzodiazepines. Flumazenil can accelerate recovery after excessive or prolonged remimazolam sedation, but re-sedation may occur, so reversal should not substitute for conservative dosing or continued monitoring (14).

## 3 Proposed mechanisms: evidence hierarchy and translational limits

Mechanistic explanations should be considered only after distinguishing established drug action from putative links to clinical outcomes. At the pharmacological level, GABA-A receptor modulation and rapid ester hydrolysis are well characterized; at the clinical level, lower rates of hypotension than with propofol are relatively consistent. Whether these properties preserve cerebral perfusion, mitigate neuroinflammation, or prevent postoperative delirium remains untested as a causal pathway. Consequently, mechanistic plausibility should not be conflated with evidence of neurocognitive protection (6, 13, 16) (Figure 2).

**Figure 2:**
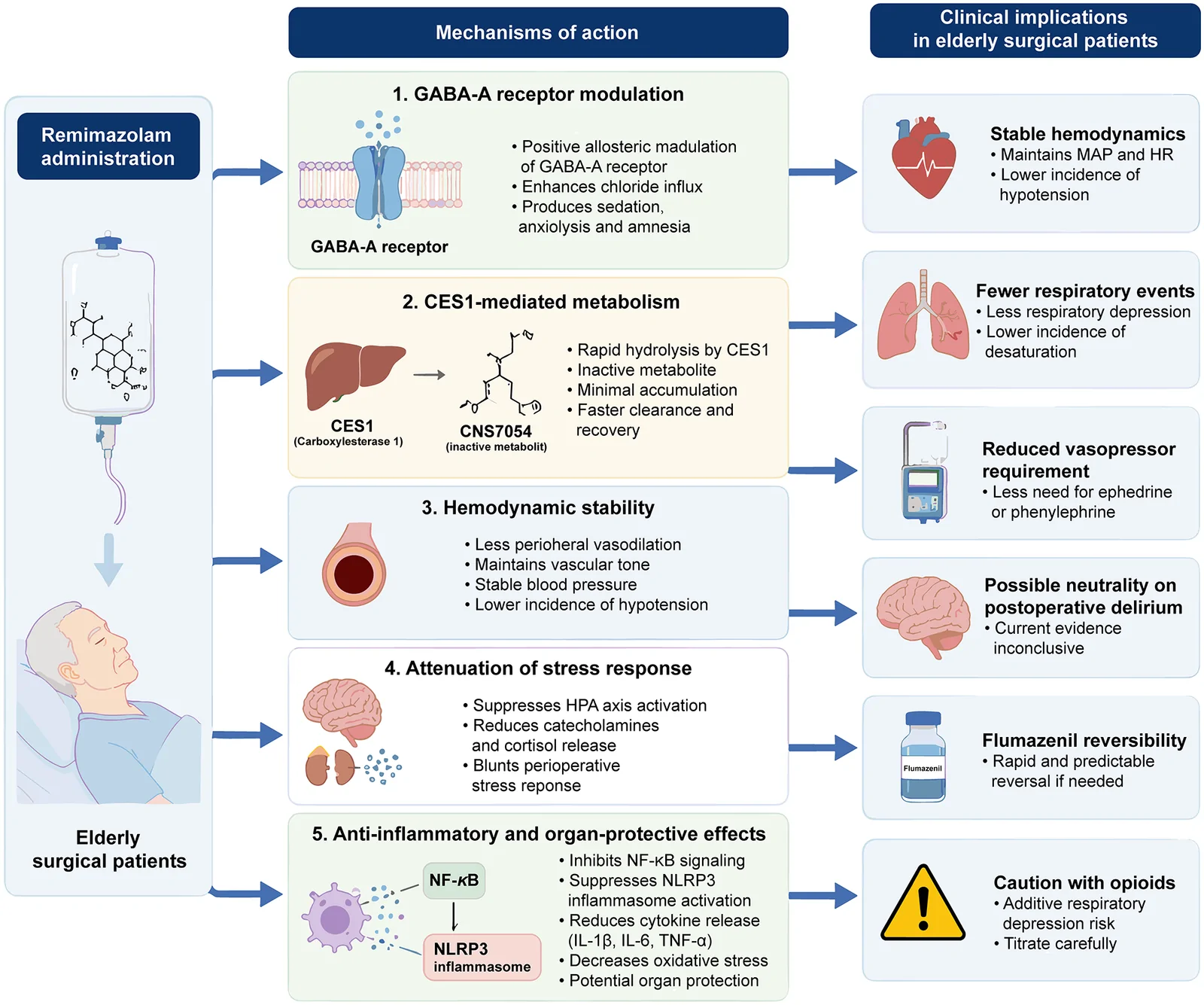
Mechanistic overview of remimazolam in older surgical patients. This schematic summarizes mechanistic pathways that may contribute to the perioperative profile of remimazolam in older surgical patients. GABA-A receptor modulation produces hypnosis, while rapid CES1-mediated metabolism limits drug accumulation. Reduced cardiovascular depression is consistent with the lower hypotension rates reported in many clinical studies. Stress-response and inflammatory pathways remain exploratory, and current evidence does not establish that these mechanisms reduce postoperative delirium. The recent lung-resection trial further emphasizes the need to distinguish emergence delirium from later POD.

A hip-replacement study reported lower postoperative catecholamine and cortisol concentrations with remimazolam, but its design could not establish a drug-specific mechanism or mediation of clinical outcomes (17). Experimental cerebral and hepatic ischemia-reperfusion models have reported changes in inflammatory or cellular stress signaling, including NF-*κ*B/NLRP3 and FOXO-related pathways (18, 19). These findings are hypothesis-generating, derive from different organs and models, and cannot be extrapolated to delirium prevention in older surgical patients without appropriately designed human studies. These mechanistic uncertainties are particularly relevant to older surgical patients, whose reduced physiological reserve and increased susceptibility to postoperative neurocognitive complications may influence the clinical balance between hemodynamic tolerability and recovery-related risks.

## 4 Why older surgical patients are a distinct population

Older adults combine reduced cardiovascular reserve with greater vulnerability to POD because frailty, multimorbidity, baseline cognitive impairment, and polypharmacy are common (1, 2). Intraoperative hypotension is associated with postoperative myocardial and renal injury, although much of this evidence is observational and does not establish that a specific hypnotic prevents end-organ injury (20). Accordingly, lower hypotension rates with remimazolam are clinically relevant safety data, but whether selecting a more hemodynamically stable hypnotic improves renal, cardiac, or neurocognitive outcomes remains unproven.

## 5 Clinical evidence in older surgical patients: randomized trials

### 5.1 Randomized evidence overview and hemodynamic profile

Across randomized surgical studies, fewer hypotensive events are the most consistent comparative signal favoring remimazolam, although effect size varies with induction strategy, maintenance regimen, surgical population, and the definition of hypotension (10–12, 21). Delirium findings are substantially less consistent. Table 1 therefore compares the major randomized trials by population, anesthetic protocol, delirium assessment window, hemodynamic and respiratory findings, and key limitations.

**Table 1:** Major randomized trials comparing remimazolam with propofol in older surgical patients.

Study/trial design/sample	Age eligibility/surgical setting	Anesthetic comparison	Delirium assessment and findings	Hemodynamic findings/reporting caveats
Yang et al. (24) Comparative RCT 320 randomized; 300 analyzed	>60 y; orthopedic surgery	Remimazolam 0.2–0.3 mg/kg vs. propofol 1.0–1.5 mg/kg for induction; balanced anesthesia and depth monitoring	CAM; POD days 1–3: 15.6% vs. 12.4% (not significant); PACU delirium not separately analyzed	Less hypotension with remimazolam; recovery metrics longer; single-center study
Fang et al. (22) (REMPPOD) Non-inferiority RCT 728 randomized	60–90 y; hip surgery	Remimazolam 0.2–0.25 mg/kg vs. propofol 1.5–2.0 mg/kg for induction; study-drug maintenance; opioids; BIS 45–60; fascia iliaca block	3D-CAM/CAM-ICU; POD days 1–3: 11.5% vs. 10.4%; non-inferiority met for prespecified window	Lower hypotension and PACU agitation; PACU delirium not part of primary POD endpoint
Cai et al. (25) Comparative RCT *n* = 136 reported	≥65 y; frailty score ≥6; hip surgery	Remimazolam 0.2–0.3 mg/kg induction and 0.3–1.0 mg/kg/h maintenance vs. propofol 1.0–1.5 mg/kg and 4–12 mg/kg/h; processed EEG	3D-CAM twice daily for 3 postoperative days; POD: 4.4% vs. 17.6% (RR 0.25)	Less post-induction hypotension and EEG burst suppression; small, selected, single-center cohort
Sim et al. (27) Non-inferiority RCT 461 randomized; 432 analyzed	≥65 y; gastrectomy	Remimazolam 1–2 mg/kg/h vs. propofol target-controlled infusion for maintenance; BIS 40–60	CAM within 72 h; POD: 7.9% vs. 7.9%; non-inferiority design	Early PACU delirium not evaluated as a separate endpoint; limited to gastrectomy
Zhang et al. (23) Comparative RCT 370 randomized	65 to <90 y; major abdominal surgery >2 h	Remimazolam 0.1–0.3 mg/kg vs. propofol 1–3 mg/kg induction; BIS-guided maintenance	CAM/CAM-ICU through day 5; per-protocol POD: 17.1% vs. 19.7% (not significant)	Less hypotension with remimazolam; single center; age-subgroup interaction exploratory
Wang et al. (13) Non-inferiority RCT 455 modified intention-to-treat	≥65 y; lung resection	Protocol-based remimazolam vs. propofol general anesthesia; delirium assessors blinded	CAM in PACU and twice daily on days 1–3; overall 48% vs. 31%; PACU 46% vs. 28%; after PACU 4% vs. 5%; non-inferiority not met	Excess risk concentrated in PACU emergence; single surgical population; respiratory endpoints not harmonized

BIS, bispectral index; CAM, Confusion Assessment Method; CAM-ICU, CAM for the ICU; 3D-CAM, 3-Minute Diagnostic Interview for CAM; EEG, electroencephalography; PACU, post-anesthesia care unit; POD, postoperative delirium; RCT, randomized controlled trial; RR, risk ratio. Outcome percentages are remimazolam versus propofol. Age entries describe eligibility criteria. Respiratory events were not uniformly defined or reported across the studies.

The distinction between an intermediate physiological outcome and a patient-centered neurocognitive outcome is important. Several trials report less hypotension with remimazolam without a reduction in POD, and the 2026 lung-resection trial reported more emergence delirium despite remimazolam's generally favorable cardiovascular profile (13, 22, 23). Hemodynamic stability and neurocognitive outcomes should therefore be interpreted as separate clinical observations unless a causal relationship is demonstrated.

Table 1 summarizes the major randomized trials comparing remimazolam with propofol in older surgical patients, highlighting study design, sample size, age eligibility, surgical setting, anesthetic protocols, delirium assessment windows, and principal clinical findings.

### 5.2 Orthopedic and hip surgery

Orthopedic and hip-surgery trials illustrate the heterogeneity of the neurocognitive evidence. Yang et al. found no significant difference in POD over postoperative days 1–3 despite fewer hypotensive events with remimazolam (24). In the multicenter REMPPOD trial, POD from postoperative day 1 to day 3 was similar with remimazolam and propofol (11.5% vs. 10.4%), while PACU agitation and hypotension were lower with remimazolam (22). By contrast, a smaller trial restricted to frail hip-surgery patients reported less POD with remimazolam (4.4% vs. 17.6%) together with less post-induction hypotension and EEG burst suppression (25). The latter result is clinically interesting but population-specific and requires replication before supporting a general delirium-preventive claim.

### 5.3 Abdominal and thoracic surgery

Abdominal-surgery trials have generally reported neutral POD findings. Delirium did not differ significantly after colorectal surgery, gastrectomy, or major abdominal surgery, although the 2026 abdominal-surgery trial again reported less hypotension with remimazolam (23, 26, 27). This recurring dissociation argues against assuming that improved hemodynamics necessarily produce lower POD incidence.

Thoracic surgery provides the strongest cautionary evidence. Earlier pulmonary-lobectomy work suggested favorable short-term cognitive and hemodynamic profiles but was not designed to establish POD prevention (28). In a subsequent randomized non-inferiority trial involving older patients undergoing lung resection, Wang et al. (13) reported findings from a modified intention-to-treat population of 455 patients. Overall delirium was more frequent with remimazolam than with propofol (48% vs. 31%), primarily owing to a higher incidence of emergence delirium in the PACU (46% vs. 28%), whereas delirium after PACU discharge through postoperative day 3 was uncommon and similar between groups (4% vs. 5%) (13). The assessment window is therefore central to interpretation.

## 6 Discussion

### 6.1 Reconciling heterogeneous trial and meta-analytic evidence

The most consequential finding of this review is a divergence between a comparatively consistent reduction in hypotension and heterogeneous neurocognitive outcomes. The pattern of delirium differs across older patients undergoing orthopedic, hip, abdominal, and thoracic surgery: several randomized trials report similar postoperative delirium (POD) rates, one relatively small frail hip-surgery trial suggests benefit, and a recent lung-resection trial reports an unfavorable signal concentrated during early emergence (13, 22–25, 27). These differences should be interpreted in terms of population, outcome definition, and study design, rather than simply as inconsistent estimates of one universal drug effect.

The evidence syntheses require the same caution. Two 2025 meta-analyses of older surgical populations found no statistically significant reduction in POD with remimazolam despite less hypotension, whereas a broader adult meta-analysis reported a lower pooled delirium risk (10, 11, 29). A later geriatric synthesis also found no POD reduction but searched only through February 2026 (12). Thus these pooled estimates predated the Wang lung-resection trial published in July 2026. Their results remain informative for their included studies, but they cannot determine how the new PACU signal affects a harmonized estimate.

Differences in ascertainment windows are central. In REMPPOD, the primary POD outcome covered postoperative days 1–3, whereas Wang et al. assessed delirium in the post-anesthesia care unit (PACU) and then twice daily for three postoperative days (13, 22). If surveillance starts the following morning, recovery-room delirium may be missed. The patient mix also differs markedly: frailty, baseline cognition, surgical stress, one-lung ventilation, processed-EEG targets, opioid exposure, regional anesthesia, and flumazenil use vary across studies. Existing trials have generally not been powered to establish which of these factors truly modifies the effect of remimazolam on delirium.

Consensus recommendations for perioperative neurocognitive nomenclature distinguish acute postoperative delirium from delayed neurocognitive recovery and longer-term postoperative neurocognitive disorders. This distinction reinforces the need to specify phenotype and observation window rather than treating every postoperative cognitive finding as one interchangeable endpoint (30).

Trial objectives are also important. A non-inferiority result does not demonstrate superiority. REMPPOD met its non-inferiority criterion for POD in the assessed postoperative-day window (22). By contrast, the lung-resection non-inferiority trial prespecified a 10% absolute margin and observed overall delirium in 48% with remimazolam versus 31% with propofol, corresponding to an absolute difference of 16.5% (95% CI, 7.7%–25.3%) and a risk ratio of 1.53 (95% CI, 1.21–1.94); non-inferiority was not established (13). Although failure to demonstrate non-inferiority is not, by itself, a formal proof of inferiority, the estimated excess emergence-delirium risk is clinically important. The trials assessed different perioperative time windows and should not be treated as interchangeable.

### 6.2 Clinical relevance of delirium in the PACU

Delirium detected immediately after anesthesia must not be dismissed merely because its symptoms may resolve before the next-day assessment. In the lung-resection trial, emergence delirium was detected in 46% of patients allocated to remimazolam compared with 28% allocated to propofol (risk ratio 1.65), whereas delirium after PACU discharge through postoperative day 3 was infrequent and similar (4% vs. 5%) (13). This finding identifies the early recovery period as the main source of the observed between-group difference, without proving that every episode progressed to persistent neurocognitive impairment.

Importantly, the clinical relevance of PACU delirium extends beyond its immediate presentation. In an observational cohort of 626 patients, Hesse et al. (31) identified PACU delirium in 125 patients and reported its association with subsequent hospital readmission (odds ratio 2.17, 95% CI 1.13–4.17) and approximately twice the likelihood of a hospital stay exceeding six days. Card et al. (32) also documented the evolution of delirium signs during PACU recovery, illustrating why a single later assessment cannot characterize this early phenotype. These studies support PACU delirium as a meaningful marker of perioperative vulnerability and adverse outcomes; they do not establish that a short PACU episode causes long-term cognitive decline, nor do they establish a causal effect of remimazolam on readmission or length of stay.

More recent evidence strengthens this prognostic context. In a retrospective cohort of 3,781 patients aged at least 70 years undergoing noncardiac, noncranial surgery, PACU delirium was detected in 9.1% and was associated with subsequent ward delirium (24.6% vs. 4.3%; adjusted odds ratio 4.4, 95% CI 3.2–6.3), as well as falls and longer hospitalization (33). Because this was an observational study, its findings support early risk identification and follow-up, not a causal claim that PACU delirium produces these outcomes or that a particular anesthetic changes their incidence.

Longer-term studies also link postoperative delirium with subsequent cognitive trajectories: a cardiac-surgery cohort found delayed cognitive recovery after delirium during the first postoperative year (34), and a separate study of older patients undergoing major elective surgery associated postoperative delirium with faster cognitive decline over six years (35). These studies concern postoperative delirium more broadly; they must not be used as direct evidence that an isolated, transient PACU episode causes persistent cognitive impairment.

For clinicians and future investigators, PACU delirium and later POD should therefore be reported separately, preferably using validated tools at prespecified time points and clearly documented timing relative to anesthesia cessation and discharge from the recovery unit. Recovery-room agitation alone should not automatically be equated with instrument-confirmed delirium. Even when later POD rates are similar, a higher burden of PACU delirium may justify closer observation, careful evaluation of reversible contributors, and planned follow-up; whether these responses improve longer-term outcomes remains to be tested.

### 6.3 Hemodynamic benefit is not a surrogate for delirium prevention

Across comparative trials and meta-analyses, remimazolam generally causes less hypotension than propofol (10, 11, 16). This advantage is meaningful for older patients with reduced cardiovascular reserve. However, it is an intermediate physiological outcome, not evidence that remimazolam prevents POD or organ injury. Neutral POD findings in several trials and the unfavorable PACU emergence-delirium signal in lung resection are inconsistent with a simple causal pathway from fewer hypotensive episodes to less delirium (13, 22, 23). Studies designed to test mediation by hypotension burden or cerebral perfusion would be required before any such claim could be made.

The wider anesthesia literature further cautions against assuming that a plausible intraoperative target automatically improves neurocognitive outcomes. In the ENGAGES randomized trial of 1,232 adults aged at least 60 years undergoing major surgery, EEG-guided anesthetic administration did not significantly reduce postoperative delirium compared with usual care (36). Although this trial did not compare remimazolam and propofol, it illustrates why a direct delirium endpoint is required before making a preventive claim.

Anesthetic choice should therefore follow an individualized trade-off framework. Remimazolam may be reasonable when propofol-associated hypotension is a major concern or benzodiazepine reversibility is advantageous, but clinicians should not select it routinely for delirium prevention. Frailty, hepatic dysfunction, baseline cognitive vulnerability, opioid exposure, and the consequences of disturbed emergence deserve attention. Conservative titration, respiratory and hemodynamic monitoring, appropriate anesthetic-depth management, and continued observation after flumazenil remain necessary (7, 14, 15). Delirium prevention should remain multimodal, including adequate analgesia, oxygenation, medication review, sleep preservation, mobilization, and structured screening (2).

### 6.4 Evidence limitations and priorities for future research

Interpretation is limited by disparate definitions of older age and frailty, incomplete characterization of baseline cognitive status, heterogeneous anesthetic and analgesic regimens, and variable delirium instruments and assessment schedules. The prominence of PACU delirium in the lung-resection study may reflect true context-specific risk and/or ascertainment differences; existing evidence cannot partition these explanations. In addition, most studies were not designed to evaluate 30-day readmission, prolonged disability, persistent cognitive change, or the causal pathways linking early delirium to those outcomes. Observational findings from Hesse et al. should therefore be viewed as prognostic associations rather than intervention effects (13, 31).

Future multicenter trials should prespecify PACU delirium and later POD as separate endpoints, apply repeated validated assessments, report frailty and baseline cognition, and document anesthetic dose, processed-EEG metrics, burst suppression, hypotension burden, opioid and dexmedetomidine exposure, regional techniques, and flumazenil use. Longer-term patient-centered outcomes—including hospital stay, readmission, functional recovery, and cognition—would clarify whether differences observed during emergence have durable clinical implications. Future meta-analyses should stratify by assessment window and surgical setting rather than pooling disparate definitions under a single POD label.

## 7 Conclusion

Remimazolam is an ultra-short-acting benzodiazepine with rapid ester-mediated metabolism and a generally favorable hemodynamic profile. Among older surgical patients, the most reproducible comparative finding is less hypotension than with propofol; delirium outcomes remain heterogeneous. Several randomized trials found similar later POD rates, one frail hip-surgery trial suggested benefit, and a 2026 lung-resection trial found more overall delirium driven predominantly by events during PACU emergence. Because PACU delirium has been associated with readmission and longer hospitalization in observational research, early recovery outcomes should be assessed and reported distinctly, without assuming that these associations are causal. Remimazolam should not be presented as a delirium-preventive drug; anesthetic choice should remain individualized while better designed trials clarify short- and longer-term neurocognitive effects.
